# Shelf-stable electrophilic trifluoromethylating reagents: A brief historical perspective

**DOI:** 10.3762/bjoc.6.65

**Published:** 2010-06-16

**Authors:** Norio Shibata, Andrej Matsnev, Dominique Cahard

**Affiliations:** 1 Department of Frontier Materials, Graduate School of Engineering, Nagoya Institute of Technology, Gokiso, Showa-ku, Nagoya 466-8555, Japan; 2 UMR 6014 CNRS – C.O.B.R.A. Université et INSA de Rouen, 1 rue Tesnière, 76130 Mont Saint Aignan, France

**Keywords:** asymmetric synthesis, electrophilic, fluorine, reagent, trifluoromethylation

## Abstract

Since the discovery by Yagupolskii and co-workers that *S*-trifluoromethyl diarylsulfonium salts are effective for the trifluoromethylation of thiophenolates, the design and synthesis of electrophilic trifluoromethylating reagents have been extensively researched in both academia and industry, due to the significant unique features that trifluoromethylated compounds have in pharmaceuticals, agricultural chemicals, and functional materials. Several effective reagents have been developed by the groups of Yagupolskii, Umemoto, Shreeve, Adachi, Magnier, Togni and Shibata. Due to the high stability and reactivity of these reagents, a series of Umemoto reagents, Togni reagent and Shibata reagent are now commercially available. In this review, we wish to briefly provide a historical perspective of the development of so-called “shelf-stable electrophilic trifluoromethylating reagents”, although this field is in constant development.

## Review

The chemistry of fluoro-organic compounds is one of the areas of the life sciences that have developed most rapidly over the last 50 years, despite the fact that fluorine is “foreign” to the organic chemistry of life since not more than a dozen of compounds containing fluorine atom(s) have been found in nature [1–2]. It is a gross understatement to say that introduction of fluorine into organic molecules often leads to significant changes in their physical, chemical and biological properties [3]. The specific physical and chemical properties of fluorine in fluorine containing compounds, especially its strong electronegativity, lipophilicity and reaction ability, differ dramatically from those of other halogens and thus lead to changes in the interaction between the molecule and components in the surrounding biological environment [4]. Fluorine has now a prestigious position especially in the design of biologically active compounds, and indeed, nearly 20% of human medicines and 35% of agrochemicals on the market contain one or more fluorine atoms [5]. Among the increasingly powerful methods that have been developed for the direct introduction of fluorine into organic compounds, trifluoromethylation is one of the most direct and straightforward strategies in the synthesis of fluorine-containing organic compounds. Efficient transfer of the trifluoromethyl group from a reagent to a target molecule is key for the reaction, and the reagents are classified according to their radical, nucleophilic or electrophilic character. Radical trifluoromethylation can be achieved from various sources of trifluoromethyl radicals that include trifluoromethyl iodide, trifluoromethylacetyl and trifluoromethylsulfonyl derivatives, *S*-trifluoromethyl xanthates and others [6]. These reagents are well-suited for trifluoromethylation of aromatics, heteroaromatics and unsaturated double bonds [7]. Nucleophilic trifluoromethylation probably represents the most versatile and actively studied methodology available for the purpose of direct trifluoromethylation. The success of this methodology is greatly indebted to the availability of the reagents. The best known reagent for nucleophilic trifluoromethylation is “Ruppert’s reagent”, trifluoromethyltrimethylsilane (Me_3_SiCF_3_), which, under catalysis, produces a trifluoromethyl anion capable of reacting with various electrophiles [6,8–10]. Despite the fact that the idea of a reagent in which the perfluoroalkyl group could be positively charged would appear at first to be nonsensical, in 1984 Yagupolskii and co-workers discovered that *S*-(trifluoromethyl)diarylsulfonium salts are effective for the electrophilic trifluoromethylation of thiophenolates. Since this pioneering work, the design and synthesis of electrophilic trifluoromethylating reagents have been extensively investigated. Historically, the chalcogenium salts developed by Umemoto and co-workers are the most widely used reagents for effective trifluoromethylation of a wide range of nucleophiles. Typical reagents are the *S*-(trifluoromethyl)dibenzothiophenium tetrafluoroborate and triflate, both of which are commercially available. More recently, in 2006, Togni and co-workers reported a new family of hypervalent iodine(III)-CF_3_ reagents as mild electrophilic trifluoromethylating agents suitable for reactions with carbon- and heteroatom-centered nucleophiles. These reagents further demonstrated generality in trifluoromethylation of a wide range of nucleophiles including the trifluoromethylation of aliphatic alcohols and these are now commercially available. In 2008, we reported a novel fluorinated Johnson-type reagent for electrophilic trifluoromethylation of carbon-centered nucleophiles. This reagent has demonstrated high efficiency in trifluoromethylation of cyclic β-ketoesters and dicyanoalkylidenes and is now commercially available. We also disclosed an easy-access to extended Yagupolskii-Umemoto type reagents, *S*-(trifluoromethyl)thiophenium salts, through triflic acid-catalyzed intramolecular cyclization of *o*-ethynylaryltrifluoromethylsulfanes. A series of *S*-(trifluoromethyl)benzo[*b*]thiophenium salts have also demonstrated high ability for trifluoromethylation of β-ketoesters and dicyanoalkylidenes to yield the trifluoromethylated products with a quaternary carbon center, even if the substrates have a rather unreactive acyclic system. In this review, we wish to briefly provide a historical perspective of the development of so-called “shelf-stable electrophilic trifluoromethylating reagents”, although, as noted in the introduction, this field is in constant development.

### First electrophilic trifluoromethylating reagent

**Scheme 1:**
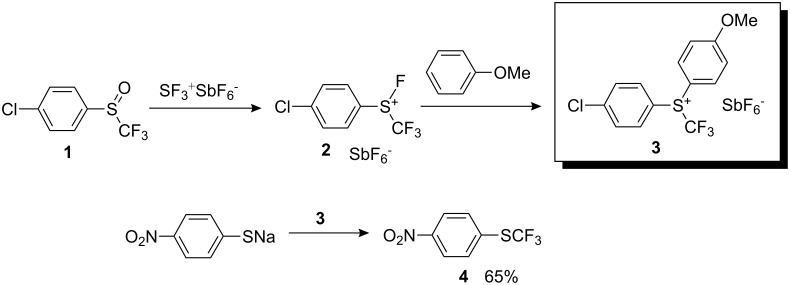
Preparation of the first electrophilic trifluoromethylating reagent and its reaction with a thiophenolate.

In 1984, Yagupolskii and co-workers successfully achieved electrophilic trifluoromethylation by means of a diaryl(trifluoromethyl)sulfonium salt, Ar_2_S^+^CF_3_ SbF_6_^−^ (**3**) [11]. This trifluoromethylating reagent was obtained by treatment of aryltrifluoromethyl sulfoxide **1** with SF_3_^+^ SbF_6_^−^ and subsequent reaction of the fluoro(trifluoromethyl) arylsulfonium salt **2** with electron-enriched arenes. Reagent **3** reacted with sodium *p*-nitrothiophenolate to give the corresponding trifluoromethyl sulfide **4** in 65% yield (Scheme 1). The substitution proceeded smoothly although electron-donating substituents on **3** partially neutralize the positive charge on the sulfur atom and thus significantly reduce the electrophilicity of the sulfonium moiety.

### Umemoto reagents: (Trifluoromethyl)dibenzothio-, seleno- and telluro-phenium salts

**Scheme 2:**
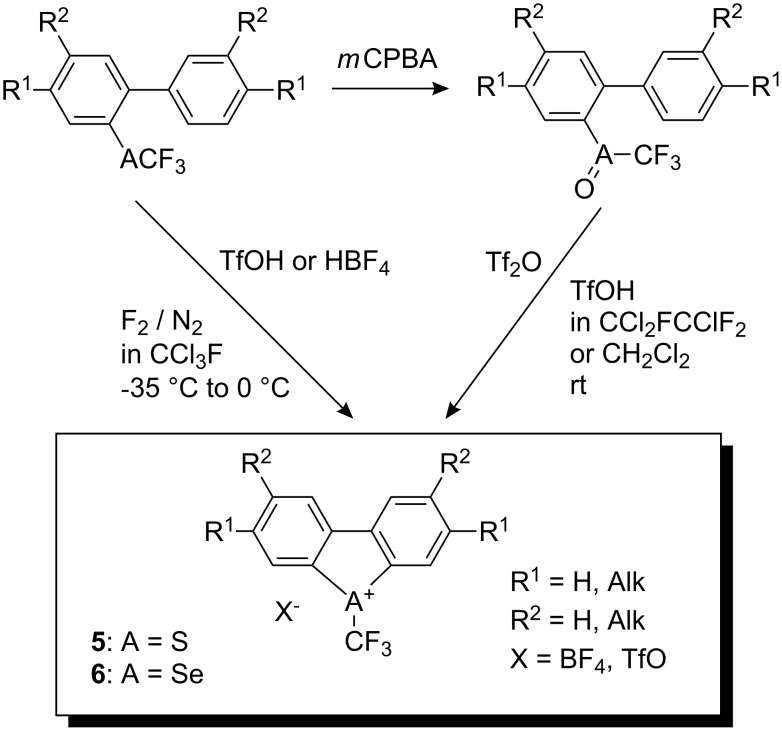
Synthetic routes to *S*-CF_3_ and *Se*-CF_3_ dibenzochalcogenium salts.

In order to find reagents with a wider scope of application, Umemoto and co-workers developed new electrophilic trifluoromethylating reagents i.e. (trifluoromethyl)dibenzoheterocyclic salts with electron-donating and electron-withdrawing substituents in benzene rings for fine tuning of their electrophilicity [12–14]. (Trifluoromethyl)dibenzothio- and selenophenium salts **5** and **6**, respectively, were synthesized either by oxidation of the starting sulfides (or selenides) with *m*-chloroperbenzoic acid followed by cyclization of the corresponding sulfoxides (or selenoxides) either with triflic anhydride or by direct fluorination with 10% F_2_/N_2_ in the presence of one equivalent of triflic acid or HBF_4_ (Scheme 2).

**Scheme 3:**
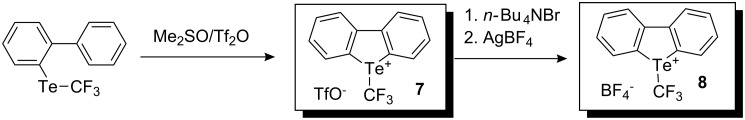
Synthesis of (trifluoromethyl)dibenzotellurophenium salts.

The tellurophenium salt **7** was synthesized in high yield by treatment of telluride starting material with an equimolar mixture of triflic anhydride and DMSO at 0 °C. Anion exchange was easily accomplished with silver tetrafluoroborate to afford **8** (Scheme 3) [13].

**Scheme 4:**
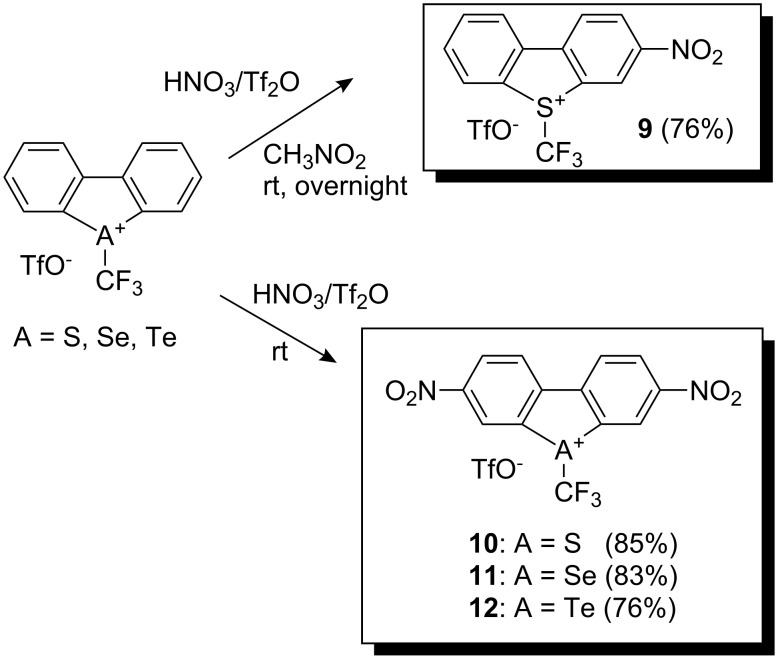
Nitration of (trifluoromethyl)dibenzochalcogenium salts.

To increase the electrophilicity of salts **5**–**8**, the salts were nitrated with nitronium triflate generated in situ from nitric acid and triflic anhydride [12]. For example, mononitro-substituted thiophenium salt **9** was obtained after overnight stirring with nitronium triflate in nitromethane at room temperature, whereas treatment for 3 days in the absence of solvent gave the dinitro-substituted thiophenium salt **10**. Similar treatment of selenophenium and tellurophenium analogs for 3 h and 1 h, respectively led to dinitro-substituted products **11** and **12** in high yields (Scheme 4).

**Scheme 5:**
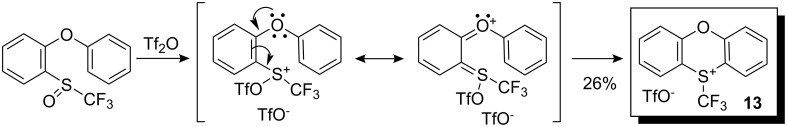
Synthesis of a sulphonium salt with a bridged oxygen.

In addition to the reagents described above, Umemoto and co-workers synthesized the phenoxathiinium salt **13** by treating 2-phenoxyphenyl trifluoromethyl sulfoxide with triflic anhydride (Scheme 5). The reaction proceeded very slowly and in low yield (6 days, 26%), presumably because of the stabilization of a cationic sulfur atom in the intermediate by the electron-donating ether moiety [13].

**Scheme 6:**
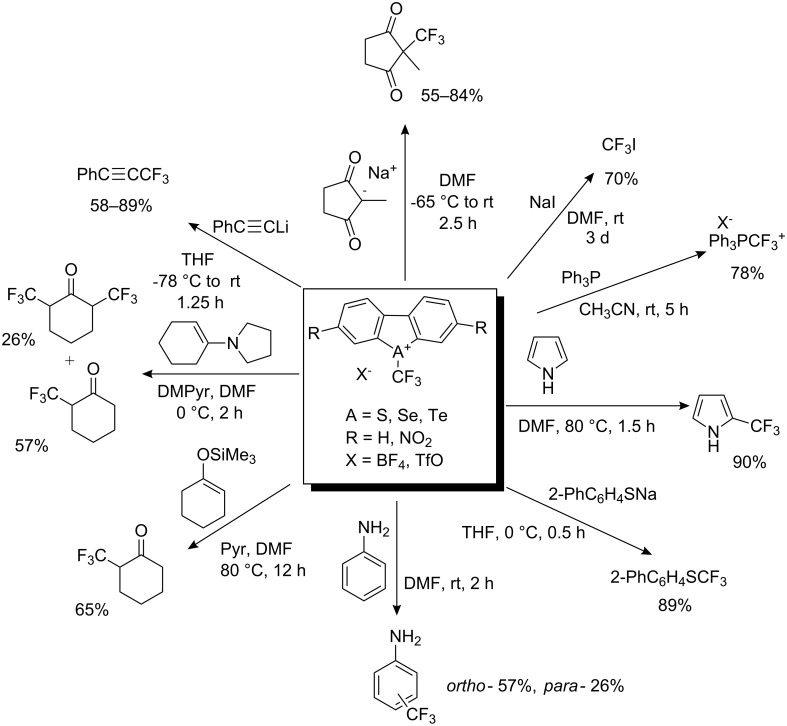
Reactivity of (trifluoromethyl)dibenzochalcogenium salts.

The relative trifluoromethylating power of chalcogenium salts increased in the order Te < Se < S while nitro-substituted reagents showed higher reactivity than non-nitrated reagents [14]. Matching the power of the trifluoromethylating agent with the nucleophile (carbanion, silyl enol ether, enamine, phenol, aniline, phosphine, thiolate) made trifluoromethylation possible as illustrated in Scheme 6.

**Scheme 7:**
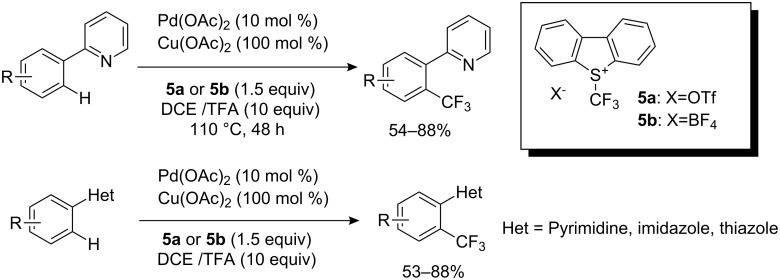
Pd(II)-Catalyzed *ortho*-trifluoromethylation of heterocycle-substituted arenes by Umemoto’s reagents.

Just prior to submission of this manuscript, an interesting paper concerning trifluoromethylation of aromatics with Umemoto reagents by Yu and co-workers appeared [15]. 2-Pyridine substituted arenes were converted to the corresponding trifluoromethylated arenes by treatment with Umemoto reagents, **5a** or **5b**, in the presence of Pd(OAc)_2_ and Cu(OAc)_2_ at 110 °C in a mixture of dichloroethane (DCE) and 10 equiv of trifluoroacetic acid (TFA). Arenes having other heterocycles such as thiazole, imidazole, or pyrimidine also reacted under the same conditions to give *ortho*-trifluoromethylated arenes in good yields (Scheme 7). Togni’s reagent (**37**, see later in the text) could be used for this reaction, although product yields were as low as 11%.

**Scheme 8:**
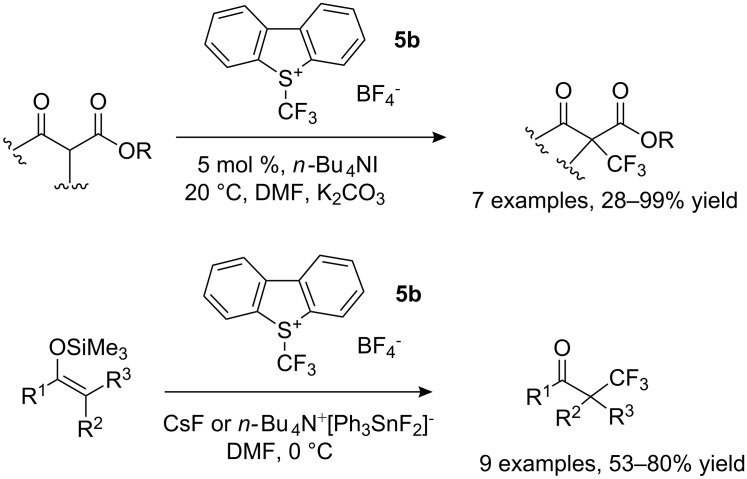
Mild electrophilic trifluoromethylation of β-ketoesters and silyl enol ethers.

The reaction conditions for trifluoromethylation of silyl enol ethers and β-ketoesters were reinvestigated by one of us (D.C.) with reagents of type **5** in order to provide milder conditions. Indeed, cyclic and acyclic β-ketoesters were efficiently trifluoromethylated with *S*-(trifluoromethyl)dibenzothiophenium tetrafluoroborate in the presence of a phase-transfer catalyst to afford the corresponding α-substituted α-trifluoromethyl β-ketoesters in good to excellent yields. In a second approach, **5** and tetrabutylammonium difluorotriphenylstannate were used for efficient electrophilic trifluoromethylation of various silyl enol ethers to give the corresponding α-trifluoromethyl ketones in good to high yields (Scheme 8) [16].

**Scheme 9:**
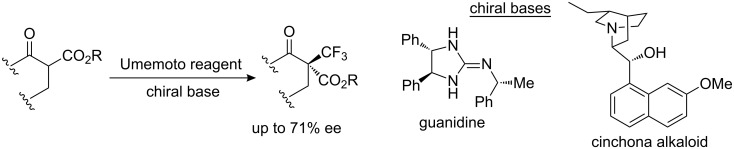
Enantioselective electrophilic trifluoromethylation of β-ketoesters.

The α-substituted α-trifluoromethyl β-ketoesters feature a stereogenic carbon center that would be interesting to control. Chiral trifluoromethylating reagents are not currently known, with the exception of compound **18** (see Scheme 14 later in the text); however, no enantioselection was observed with this reagent. Umemoto was first to report, in 1994, an enantioselective electrophilic trifluoromethylation of a ketone enolate mediated by a chiral borepin derived from a binaphthol with *S*-(trifluoromethyl)dibenzothiophenium tetrafluoroborate **5b**. The best enantiomeric excess was 45% for 20% yield [17]. In 2008–2009, we found that chiral nonracemic cinchona alkaloids and guanidines act as Brønsted bases to generate ammonium or guanidinium enolates for the enantioselective electrophilic trifluoromethylation of β-keto esters with Umemoto reagents with good enantioselectivities in the range 60–71% (Scheme 9) [18–19].

**Scheme 10:**
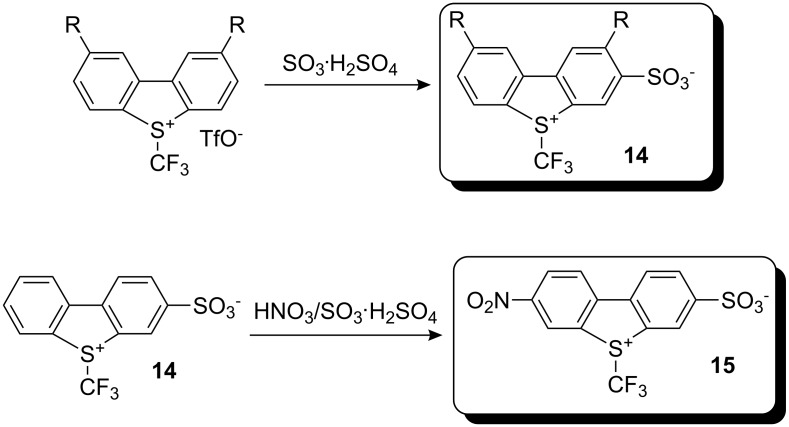
Preparation of water-soluble *S*-(trifluoromethyl)dibenzothiophenium salts.

The reagents so far described lead to by-products (dibenzothio-, seleno-, and tellurophene) after the trifluoromethylation reaction that are sometimes difficult to separate from the desired trifluoromethylated products. To overcome this drawback, Umemoto and co-workers synthesized sulfonated analogs of (trifluoromethyl)dibenzochalcogenium salts by sulfonation with fuming sulfuric acid. Further nitration of sulfonate **14** led to a more reactive nitro-substituted derivative **15** (Scheme 10). These reagents allow easy separation of by-products from the desired trifluoromethylated products by simple filtration or washing [20].

**Scheme 11:**
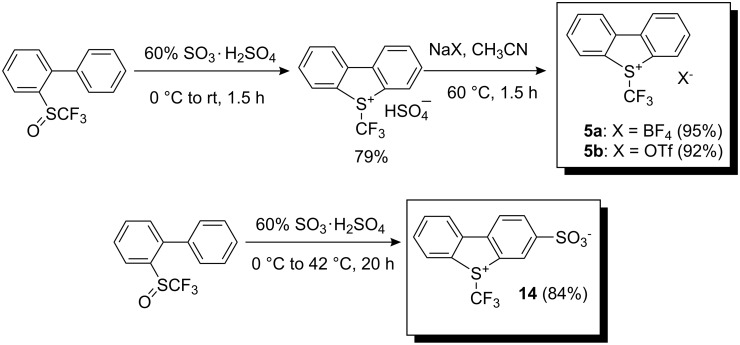
Method for large-scale preparation of *S*-(trifluoromethyl)dibenzothiophenium salts.

Since it was of interest to find an attractive synthetic method to make these reagents commercially available, Umemoto and co-workers developed a new route appropriate for the large-scale preparation of *S*-(trifluoromethyl)dibenzothiophenium salts. For instance, 2-(phenyl)phenyl trifluoromethyl sulfoxide was converted into the corresponding sulfonium salt by treatment with an excess amount of 60% SO_3_·H_2_SO_4_ at 0 °C followed by hydrogen sulfate anion exchange with tetrafluoroborate or triflate ion (Scheme 11). Increasing the reaction temperature during cyclization led to the corresponding water soluble 3-sulfonate analog **14** [21].

### Extended Yagupolskii–Umemoto-type reagents

**Scheme 12:**
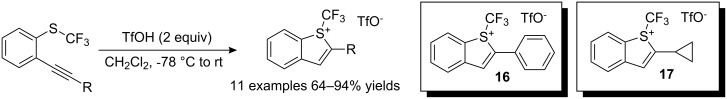
Triflic acid catalyzed synthesis of 5-(trifluoromethyl)thiophenium salts.

In 2010 a novel method for synthesis of *S*-(trifluoromethyl)sulfonium salts was developed by Shibata and co-workers. The new approach allowed access to Yagupolskii- and Umemoto-like compounds that are benzothiophenium salts rather than dibenzo analogs. *Ortho*-ethynylaryl- and alkyl-trifluoromethylsulfanes were cyclized under strong acidic conditions with triflic acid to give the corresponding sulfonium salts in 64–94% yields (Scheme 12). It should be noted, that in the presence of gold or copper salts no cyclization occurs [22].

**Scheme 13:**
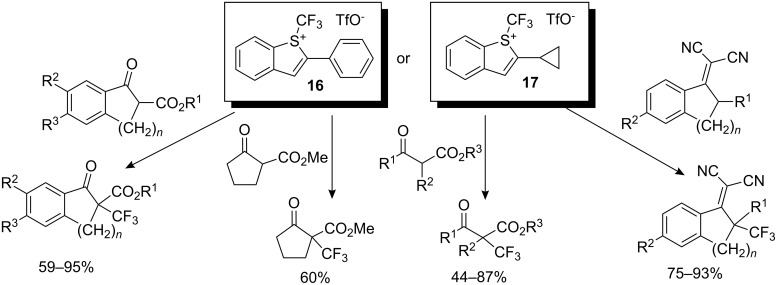
Trifluoromethylation of β-ketoesters and dicyanoalkylidenes by *S*-(trifluoromethyl)benzothiophenium salts.

A number of sulfonium salts were obtained, in particular **16** and **17**, which were evaluated as trifluoromethylating agents for β-ketoesters and dicyanoalkylidenes. The cyclopropyl-substituted reagent **17** gave slightly better yields than the phenyl-substituted reagent **16** and much higher yields than the commercially available Umemoto or Togni reagents in trifluoromethylation reactions. Of particular interest, the vinylogous trifluoromethylation of dicyanoalkylidenes afforded an access to allylic trifluoromethylated compounds (Scheme 13). All the reactions were carried out in acetonitrile at −43 °C to room temperature in the presence of a base: DBU or *tert*-butylimino-tri(pyrrolidino)phosphorane (1.2 to 2.2 equivalents).

**Scheme 14:**
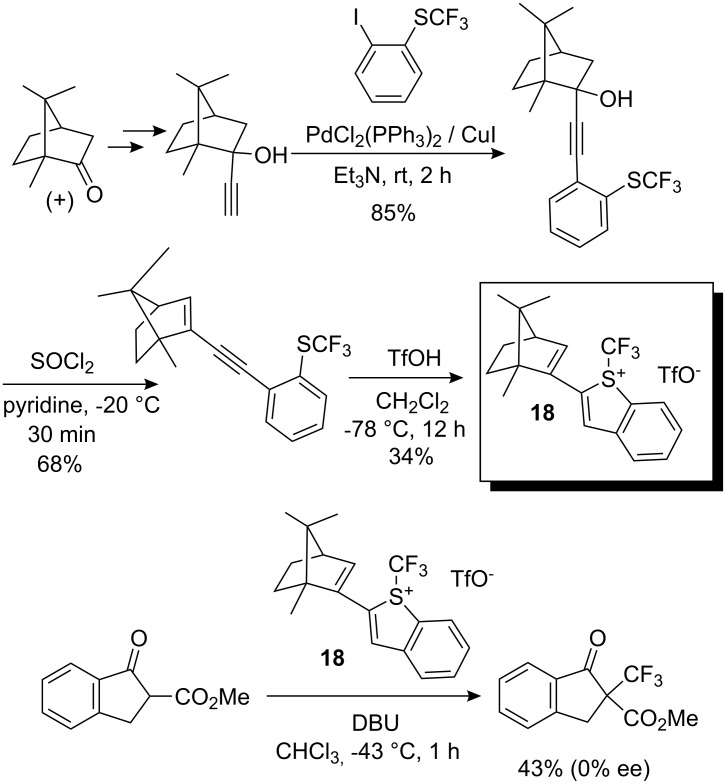
Synthesis of chiral *S*-(trifluoromethyl)benzothiophenium salt **18** and attempt of enantioselective trifluoromethylation of a β-ketoester.

One of the potential advantages of *S*-(trifluoromethyl)benzothiophenium salts is the ease with which the thiophene 2-position can be modified by chiral groups thus giving the possibility to achieve enantioselective trifluoromethylation of prochiral substrates. This is one of the important issues that is only partially solved in fluoro-organic chemistry. Therefore, chiral reagent **18** was designed and synthesized from (1*R*)-(+)-camphor as shown in Scheme 14. Reagent **18** was obtained as a 1:1 mixture of diastereoisomers originating from the chirality at the sulfur atom. The trifluoromethylation of a β-ketoester by **18** was then carried out in the presence of DBU to furnish the trifluoromethylated β-ketoester in 43% yield but as a racemate (Scheme 14) [22].

### *O*-(Trifluoromethyl)oxonium salts

**Scheme 15:**
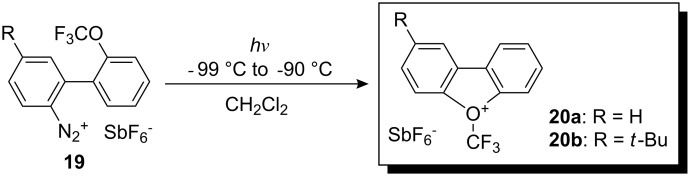
Synthesis of *O*-(trifluoromethyl)dibenzofuranium salts.

All the previously described reagents allow trifluoromethylation only of soft nucleophiles and there is no possibility of preparing *N*-CF_3_ or *O*-CF_3_ compounds by direct trifluoromethylation via these compounds. *O*-Trifluoromethyl oxonium salts were anticipated to act as a useful source of the highly electrophilic trifluoromethyl group but until the work of Umemoto and co-workers initiated in 1994 and published as a full paper in 2007 [23], their synthesis remained problematic. *O*-(Trifluoromethyl)dibenzofuranium salts **20a**,**b** are thermally unstable compounds that are obtained by photochemical decomposition at very low temperature of diazonium salts **19** (several synthetic steps are required to obtain such precursors including the construction of the CF_3_O-aryl moiety) (Scheme 15).

The trifluoromethyloxonium salts decompose to yield CF_4_ and dibenzofuran derivatives from −70 °C. Decomposition of the salts is rapid at –30 °C. However, reaction with *O*- and *N*-centered nucleophiles was possible with in situ generated trifluoromethyloxonium salts obtained by irradiation of **19** with a high-pressure mercury lamp at low temperature in dichloromethane; other chalcogenium salts lead to *C*-trifluoromethylation.

**Scheme 16:**
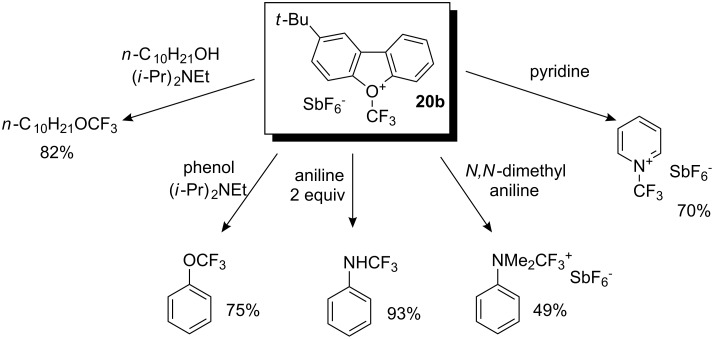
Photochemical *O*- and *N*-trifluoromethylation by **20b**.

Reagent **20b** with a *t*-Bu substituent was especially suitable for this process due to its high solubility in dichloromethane and was selected for investigating the trifluoromethylating activity in reactions with various *O*- and *N*-nucleophiles. Scheme 16 illustrates the type of substrates that could be efficiently subjected to electrophilic trifluoromethylation with **20b**. Alcohols were smoothly trifluoromethylated at low temperature in the presence of 2-chloropyridine or di(*iso*-propyl)ethylamine as an acid acceptor to give corresponding *O*-trifluoromethylated products in high yields. Primary and secondary amines also reacted with **20b** to afford *N*-CF_3_ products in good yields. Tertiary amines as well as pyridines gave quaternary ammonium and pyridinium salts, respectively.

**Scheme 17:**
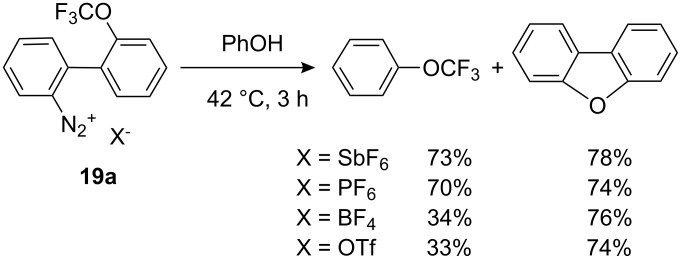
Thermal *O*-trifluoromethylation of phenol by diazonium salt **19a**. Effect of the counteranion.

Electrophilic trifluoromethylation could also be achieved by thermal decomposition of 2-(trifluoromethoxy)biphenylyl-2′-diazonium salts such as **19a** (R=H) through in situ generation of a trifluoromethyloxonium salt. The yield of trifluoromethylated products was highly dependent on the counteranion as observed in the trifluoromethylation of phenol (Scheme 17) [23].

**Scheme 18:**
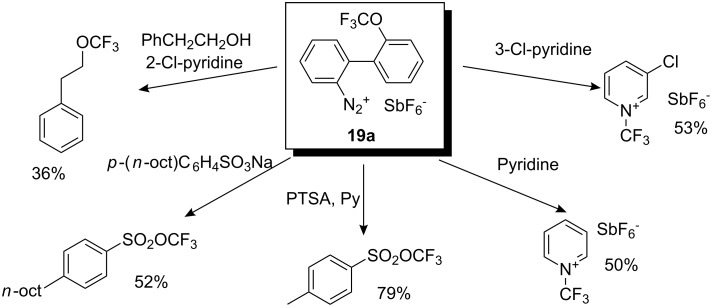
Thermal *O*- and *N*-trifluoromethylations.

The synthetic application of thermally prepared *O*-(trifluoromethyl)dibenzofuranium hexafluoroantimonate from **19a** with various nucleophiles is illustrated in Scheme 18.

In both photochemical and thermal reactions only *O*- and *N*-trifluoromethylated products were observed. *O*-CF_3_ reagents are the actual source of trifluoromethyl cation because of their ability to trifluoromethylate hard nucleophiles in contrast to other chalcogenium salts that react only with soft nucleophiles. However, this method suffers from several shortcomings, thus rendering it difficult to exploit.

### *S*-(Trifluoromethyl)diarylsulfonium salts

**Scheme 19:**
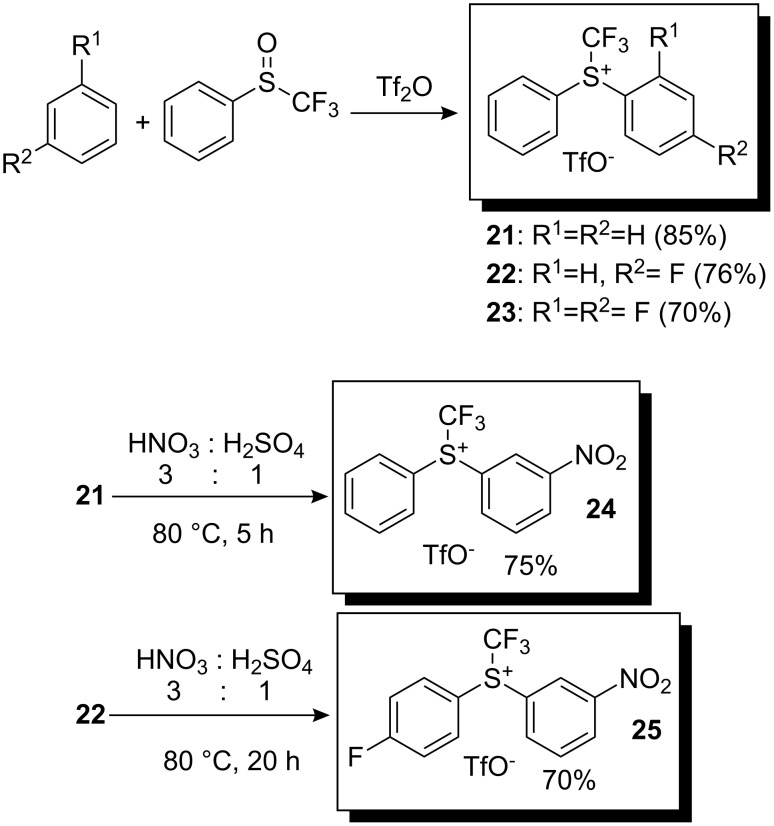
Method of preparation of *S*-(trifluoromethyl)diphenylsulfonium triflates.

In 1998, Shreeve and co-workers developed a simpler method for the preparation of Yagupolskii-type reagents i.e. non-heterocyclic trifluoromethyldiarylsulfonium triflates. By treating phenyl trifluoromethylsulfoxide with benzene or its derivatives (fluorobenzene and 1,3-difluorobenzene) in triflic anhydride at room temperature for 12 h *S*-(trifluoromethyl)diphenylsulfonium triflate **21** and its derivatives, **22** and **23**, were obtained in good yields via intermolecular condensation (Scheme 19) [24]. The products were easily purified by column chromatography and recrystallization. To increase the reactivity towards nucleophiles, a nitro group was introduced in the meta-position of the benzene ring by a conventional nitration reaction.

**Scheme 20:**
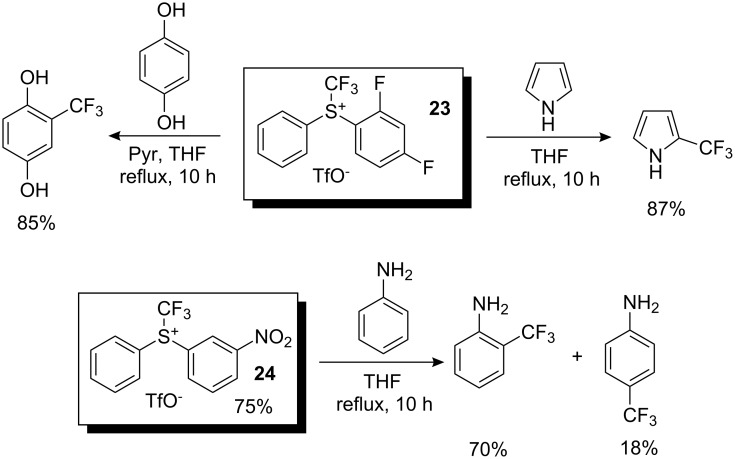
Reactivity of some *S*-(trifluoromethyl)diarylsulfonium triflates.

Shreeve and co-workers used these reagents for the trifluoromethylation of aromatic systems [24]. After optimization of reaction conditions, they found that the best result for the trifluoromethylation of aniline with reagent **24** led to a mixture of 2-trifluoromethylaniline and 4-trifluoromethylaniline in a 4:1 ratio. *p*-Hydroquinone was trifluoromethylated with **23** to produce 2-trifluoromethyl-*p*-hydroquinone in 85% yield, whereas 2-trifluoromethylpyrrole was obtained from pyrrole in 87% yield with the same trifluoromethylating reagent (Scheme 20).

**Scheme 21:**
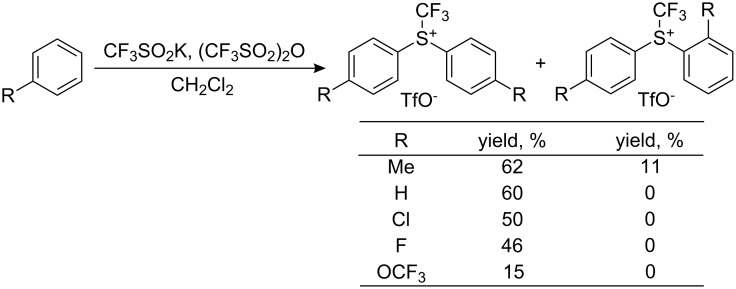
One-pot synthesis of *S*-(trifluoromethyl)diarylsulfonium triflates.

In 2006, the group of Blazejewski and Magnier presented a one-pot synthesis of Shreeve’s reagents, *S*-(trifluoromethyl)diarylsulfonium salts, by reacting an aromatic compound with potassium triflinate in triflic anhydride and dichloromethane at room temperature via the advantageous in situ formation of the aryl trifluoromethylsulfoxide (Scheme 21). The yields of the *S*-(trifluoromethyl)diarylsulfonium salts were moderate to good [25].

**Scheme 22:**
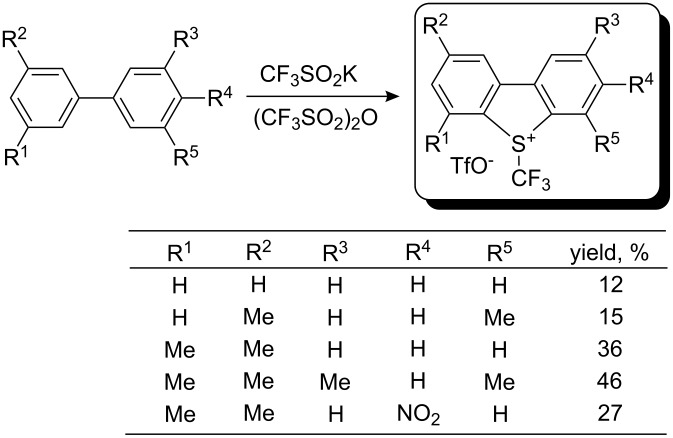
One-pot synthesis of Umemoto’s type reagents.

The same group later reported an improved experimental protocol that does not require solvent and gives better yields, up to 77% [26]. In this work, it was stressed that the purity of trifluoromethanesulfinate salts is an essential factor for the success of this reaction; low purity of the latter decreased the yield of the desired sulfonium salt. Starting from biphenyls, the method is applicable to the synthesis of Umemoto’s type reagents; yields strongly depend on the presence or absence of substituents in the aromatic rings (Scheme 22).

**Scheme 23:**
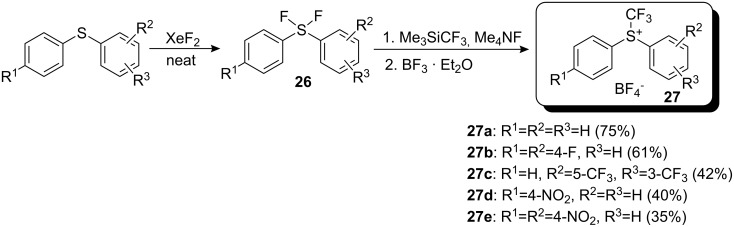
Preparation of sulfonium salts by transformation of CF_3_^−^ into CF_3_^+^.

Recently Yagupolskii and co-workers proposed a new route for the synthesis of *S*-(trifluoromethyl)diarylsulfonium salts by transformation of the nucleophilic trifluoromethylating reagent, CF_3_SiMe_3_, into an electrophilic one [27]. This method opens up the possibility for the preparation of various reagents with electron-withdrawing substituents even in the para-position of aromatic compounds. The difluorosulfurane **26**, obtained from the corresponding sulfide by treatment with xenon difluoride, was reacted first with Me_3_SiCF_3_ in the presence of fluoride ions and then with boron trifluoride to give the trifluoromethylsulfonium salt **27** (Scheme 23).

**Scheme 24:**
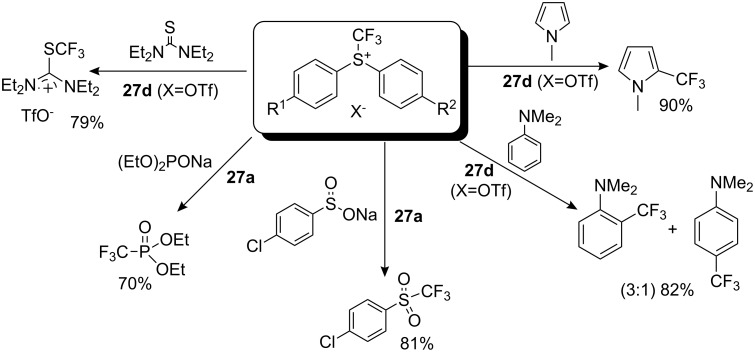
Selected reactions with the new Yagupolskii reagents.

The reactivity of these new sulfonium salts was investigated by examining their reactions with different nucleophiles (Scheme 24). Reagents **27d** and **27e** showed the best reactivity. The reaction with sodium iodide to yield CF_3_I was studied. Compound **27a** reacted only when heated, whilst the reactions with **27d** and **27e** were completed at room temperature after 6 h and 3 h, respectively. For further investigation of trifluoromethylation ability, reagents **27a** and **27d** (X=OTf or BF_4_) were selected. Reactions with *N*-methylpyrrole and *N*,*N*-dimethylaniline gave similar results to those observed using Shreeve reagents. The authors demonstrated the possibility of trifluoromethylation of sulfur-containing compounds with a partial negative charge on the sulfur atom. Thus, tetraethylthiourea reacted with **27d** (X=OTf) to give tetraethylamino(trifluoromethylthio) carbenium triflate. The reaction of **27a** (X=OTf) with sodium diethoxyphosphinate and sodium *p*-chlorophenylsulfinate led to diethyl trifluoromethanephosphonate and *p*-chlorophenyltrifluoromethylsulfone, respectively (Scheme 24).

**Scheme 25:**
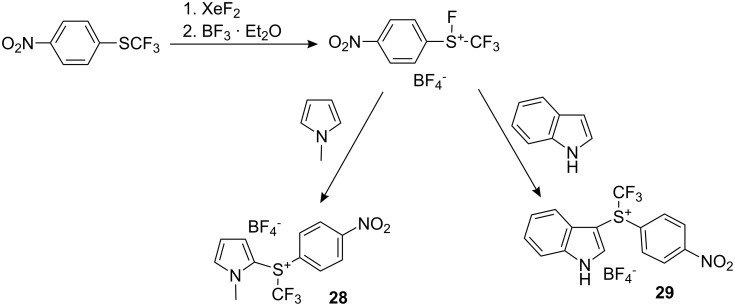
Synthesis of heteroaryl-substituted sulfonium salts.

Interestingly, the method was developed for the synthesis of heteroaromatic diarylsulfonium salts. Thus, *p*-nitrophenyl trifluoromethyl sulfide was reacted first with xenon difluoride in the absence of solvent and then with boron trifluoride. The addition of an electron-rich heterocycle gave products **28** and **29** (mixtures of 2- and 3-substituted adducts; yields not provided) (Scheme 25). However, the trifluoromethylating ability of these compounds has not yet been investigated.

### Neutral *S*–CF_3_ reagents

**Scheme 26:**
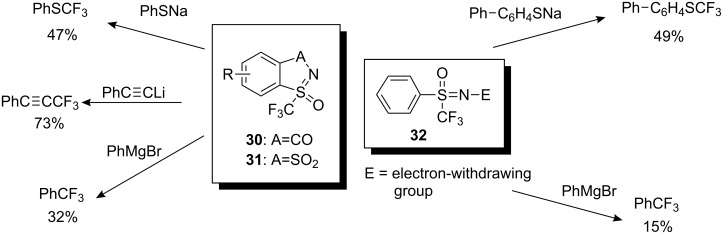
First neutral *S*-CF_3_ reagents.

The first neutral reagents for electrophilic trifluoromethylation were synthesized by Adachi and co-workers at Daikin laboratories in 2003. 1-Oxo-1-trifluoromethyl-1λ^6^-benzo[*d*]isothiazol-3-one (**30**), and 1-trifluoromethyl-benzo[1,3,2]dithiazole 1,3,3-trioxide (**31**) as well as acyclic sulfoximines **32** were synthesized as new trifluoromethylating agents (Scheme 26). It was possible to trifluoromethylate carbanions, enamines, and thiolate anions with these reagents, albeit in low to moderate yields [28].

### Neutral hypervalent iodine(III)–CF_3_ reagent

**Scheme 27:**
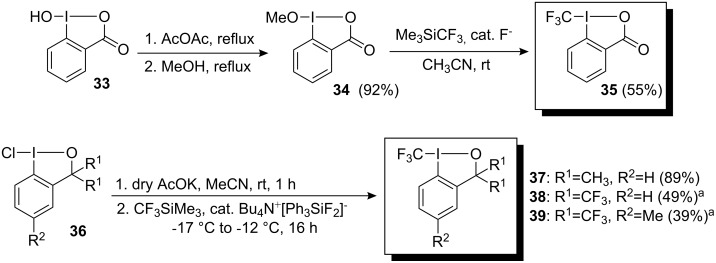
Synthesis of Togni reagents. ^a^Yield for the two-step procedure.

Initial attempts by Yagupolskii and Umemoto to synthesize iodonium salts with a trifluoromethyl group were unsuccessful. Whilst iodonium salts including *p*-tolylperfluoroalkyliodonium chlorides, perfluoroalkylphenyliodonium triflates (FITS) and perfluoroalkylphenyliodonium hydrogen sulfates (FIS) have been reported as perfluoroalkylating agents, they do not function as trifluoromethylating agents [29–31]. The reason is the required synthetic intermediates have low stability compared to the intermediates with R_f_ groups with more than one carbon atom. In 2006 Togni and co-workers reported a new family of hypervalent iodine compounds in which the CF_3_ group is bonded directly to the iodine atom. The overall synthetic protocol depends on a formal umpolung of the CF_3_ group since nucleophilic ligand displacement with CF_3_^−^ at the hypervalent iodine atom is carried out during the synthesis of these CF_3_^+^ donor reagents. For example, reaction of the methyl ester of 2-iodosylbenzoic acid **34**, with Me_3_SiCF_3_ in the presence of a catalytic amount of fluoride ions in CH_3_CN at ambient temperature gave 1-trifluoromethyl-1,2-benziodoxol-3-(1*H*)-one (**35**) in 55% yield (Scheme 27) [32]. Reagents **37**–**39** were preferentially obtained in an improved, practical, one-pot procedure by substitution of chloro substituent in **36** by an acetoxy group followed by fluoride catalyzed substitution with Ruppert’s reagent (Scheme 27) [33]. These reagents are shelf-stable, non-explosive under ambient conditions but should not be heated as solid materials.

**Scheme 28:**
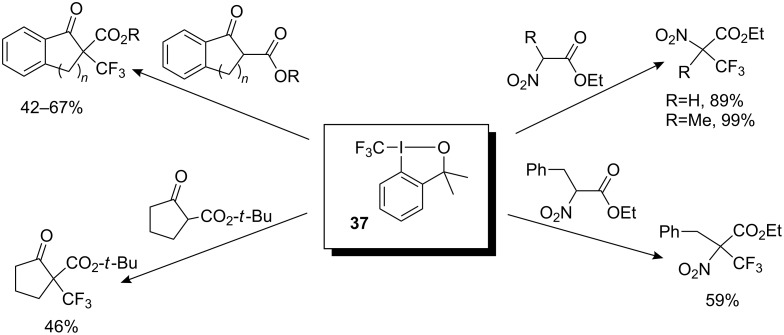
Trifluoromethylation of *C*-nucleophiles with **37**.

These new electrophilic trifluoromethylating reagents were initially evaluated in reactions with carbonyl compounds such as β-keto esters and α-nitro esters. In particular, reagent **37** was found to be an effective trifluoromethylating agent. Under phase-transfer catalysis the β-keto esters derived from indanone, tetralone and pentanone in the presence of **37** gave the corresponding trifluoromethylated product in 42–67% yields. The new reagents showed a clear advantage in the reaction with α-nitro esters; the reaction proceeded smoothly in CH_2_Cl_2_ in the presence of a catalytic amount of CuCl_2_ (Scheme 28) [33]. Interestingly, 2-(2-iodophenyl)propan-2-ol formed as by-product in the reactions of **37** with the substrates could be isolated and recycled.

**Scheme 29:**
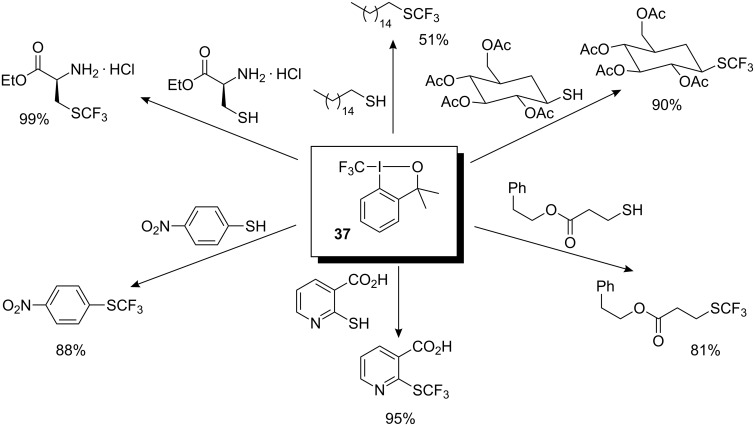
Selected examples of trifluoromethylation of *S*-nucleophiles with **37**.

The same group studied the reactivity of hypervalent iodine–CF_3_ reagents with different types of sulfur-, phosphorus- and oxygen-centered nucleophiles. Firstly, it was demonstrated that sulfur-centered nucleophiles react with hypervalent iodine–CF_3_ reagents. Thus, both aromatic and aliphatic thiols underwent *S*-trifluoromethylation smoothly in the presence of 1.1 equiv of **37** to afford the corresponding products in 51–99% yields (Scheme 29) [33]. The reaction outperforms other methods for synthesis of the SCF_3_ motif and shows high functional-group tolerance, and has particular application for the synthesis of sugar and amino acid derivatives.

**Scheme 30:**
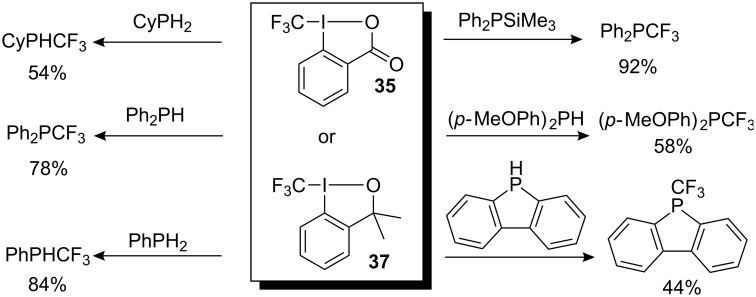
Selected examples of trifluoromethylation of *P*-nucleophiles with **35** and **37**.

Reagents **35** and **37** have been identified as mild and efficient trifluoromethylating reagents for primary and secondary aryl- and alkylphosphines. Both reacted with equal efficiency at −78 °C to rt without any added base since the reagents generate base in situ (a carboxylate from **35** and an alcoholate from **37**). *P*-Trifluoromethyl phosphines were formed in moderate to high yields from either diarylphosphines or *P*-trimethylsilylated derivatives under the same reaction conditions (Scheme 30). By contrast, the corresponding lithium and potassium phosphides (MPPh_2_) gave only trace amounts of the trifluoromethylated product [34–35].

**Scheme 31:**
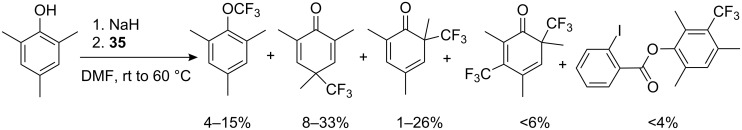
Trifluoromethylation of 2,4,6-trimethylphenol with **35**.

The reaction of phenols with reagent **35** was investigated. From 2,4,6-trimethylphenol, the expected 1,3,5-trimethyl-2-(trifluoromethoxy)benzene was obtained only in poor yields in the range 4–15%. The trifluoromethylation was carried out in the presence of sodium hydride in DMF at different temperatures and occurred preferentially at the ortho- and para-positions of the aromatic ring (Scheme 31). Other substituted phenols were used as substrates under conditions yielding only *C*-trifluoromethylated products [36].

**Scheme 32:**
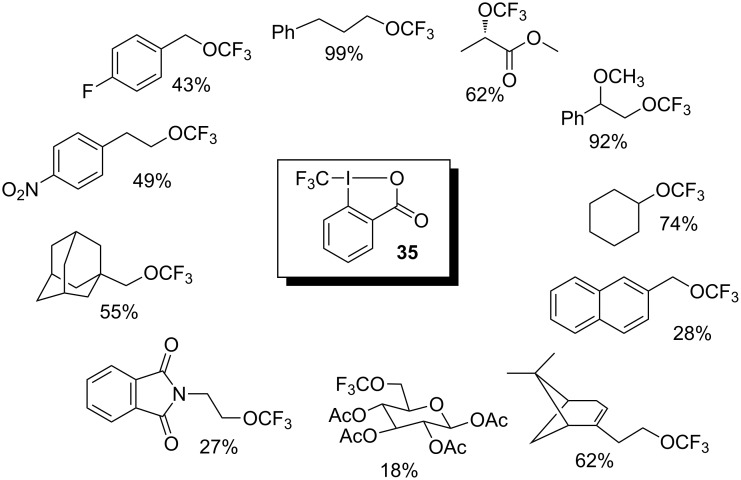
Examples of *O*-trifluoromethylation of alcohols with **35** in the presence of 1 equiv of Zn(NTf_2_)_2_.

Although *O*-trifluoromethylation of phenols still remains an unsolved problem, the trifluoromethylation of aliphatic alcohols is now possible as a result of the efforts of Togni and co-workers who discovered that the transfer of a CF_3_ group to an alcohol oxygen atom could be achieved in the presence of zinc (II) salts. Thus, 1-pentanol was quantitatively converted to the corresponding trifluoromethyl ether, which was obtained in high yield, by treatment with reagent **35** in the presence of Zn(OTf)_2_ or Zn(NTf_2_)_2_. An even higher yield of the trifluoromethyl ether resulted when the alcohol was used both as substrate and solvent in the presence of a catalytic amount of zinc salt. After optimisation of reaction conditions, different alcohols were subjected to the *O*-trifluoromethylation reaction. The reaction proceeded smoothly when primary and secondary aliphatic alcohols were used (Scheme 32). Alcohols such as *t*-BuOH, as well as phenols, could not be *O*-trifluoromethylated [37].

**Scheme 33:**
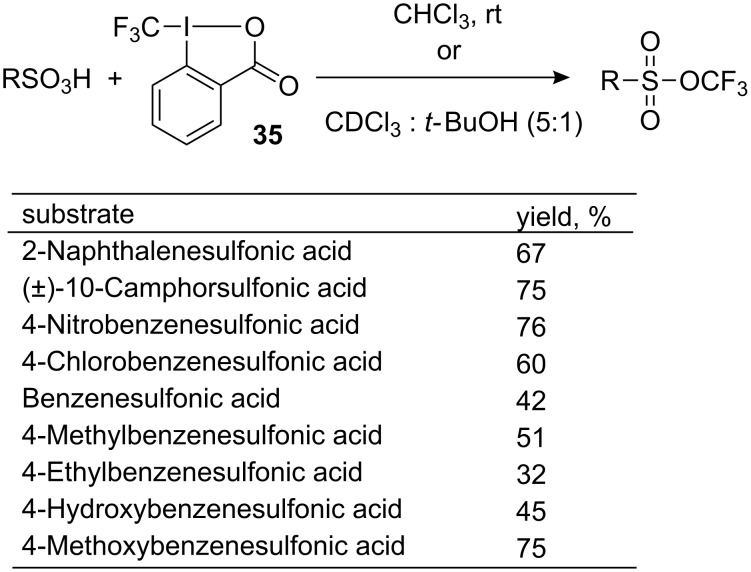
Formation of trifluoromethyl sulfonates from sulfonic acids and **35**.

Further investigations into the trifluoromethylating ability of **35**, revealed that sulfonic acids undergo *O*-trifluoromethylation to give the corresponding trifluoromethyl sulfonates in good to high yields under facile reaction conditions, i.e., in chloroform, overnight, at ambient temperature (Scheme 33). The reagent **35** is activated by the Brønsted acidity of the sulfonic acids. No reaction took place with *p*-toluenesulfonate salts or with substrates having an internal base moiety such as 4-aminobenzenesulfonic acid [38].

**Scheme 34:**
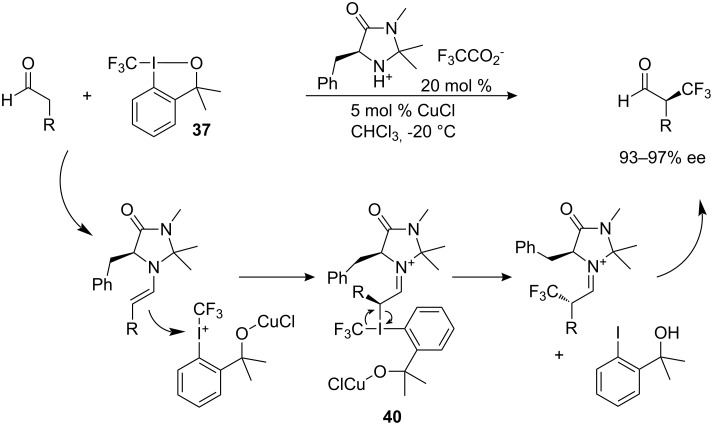
Organocatalytic α-trifluoromethylation of aldehydes with **37**.

The first highly enantioselective electrophilic trifluoromethylation of aldehydes has only very recently been reported by MacMillan by using a combination of organocatalysis with Togni’s reagent **37** [39]. This report appeared only just after a photolytic approach, also reported by MacMillan, that employs CF_3_^•^ radical generated from CF_3_I [40]. However, the reaction with bench-stable Togni’s reagent is mechanistically distinct from the previous radical approach (Scheme 34). In accord with a similar mechanism proposed by Togni [37], the resulting λ^3^-iodane species **40** undergo rapid reductive elimination with stereoretentive CF_3_ transfer. High enantioselectivities in the range 93–97% were measured for the corresponding alcohols because of post-reaction racemization of aldehydes. Although the scope of this asymmetric reaction is limited to aldehydes, the level of enantioselectivity is superior to that obtained in the enantioselective trifluoromethylation of β-ketoesters.

### Fluorinated Johnson’s type reagent

**Scheme 35:**
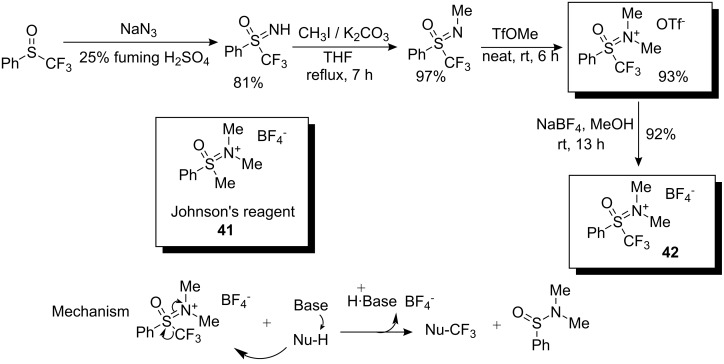
Synthesis of reagent **42** and mechanism of trifluoromethylation.

In 2008, a novel type of electrophilic trifluoromethylating agent, a trifluoro analog of Johnson’s methyl-transfer reagent **41**, was synthesized by Shibata and co-workers. Transfer of the CF_3_ group from **42** to various substrates proceeds via nucleophilic attack at the CF_3_ group with elimination of *N*,*N*-dimethylbenzenesulfinamide. The synthetic route to the sulfoximinium salt **42** starts from phenyl trifluoromethyl sulfoxide as depicted in Scheme 35. The triflate salt is an ionic liquid at room temperature whereas the tetrafluoroborate **42** is obtained as colorless crystals.

**Scheme 36:**
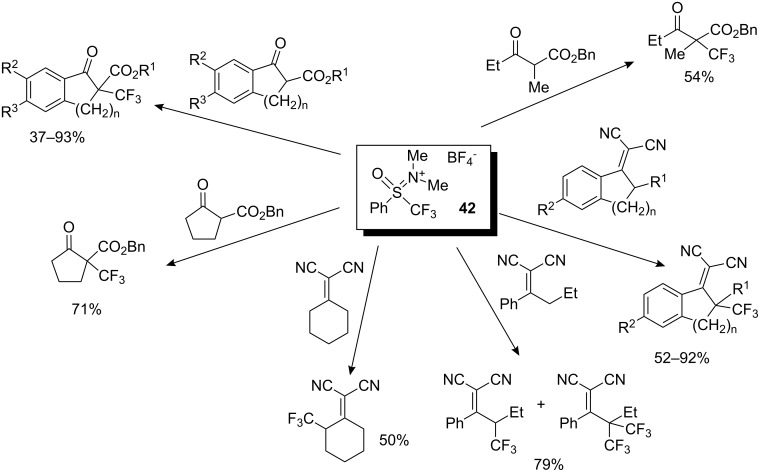
Trifluoromethylation of β-ketoesters and dicyanoalkylidenes with **42**.

An initial series of experiments to optimize the reaction conditions for efficient trifluoromethyl group transfer to indanone carboxylate were found to be the use of DBU as base and dichloromethane as solvent at room temperature. Guanidine bases, such as TMG or TBD, and phosphazene bases P^1^-*t*-Bu or P^2^-Et were equally effective. The trifluoromethylation reaction did not take place with either pyridine or triethylamine. Transfer of trifluoromethyl group from **42** to various β-ketoesters and dicyanoalkylidenes was investigated. β-Ketoesters such as indanone, tetralone and oxocyclopentane carboxylates gave the corresponding trifluoromethylated products in 52–92% yields (Scheme 36). In the case of an acyclic ester, a good yield was achieved only in the presence of the phosphazene base P^2^-Et. When the reaction was carried out in the presence of nitrobenzene there was no decrease in the yield of the desired product. Consequently, this process was classified as electrophilic trifluoromethylation. The first example of vinylogous trifluoromethylation of dicyanoalkylidenes was also reported with reagent **42** (Scheme 36). The corresponding trifluoromethylated dicyanoalkylidenes were obtained in good to high yields (50–92%) preferentially in the presence of P^1^-*t*-Bu as base in CH_2_Cl_2_ at room temperature for 1 h [41]. Reagent **42** is now commercially available in Japan.

## Conclusion

We have discussed the synthesis and reactivity of shelf-stable electrophilic trifluoromethylating reagents. Since the initial report in 1984 this field has been increasingly active, however, there are still high challenges due to the current limitations of these reagents. A broader substrate scope is highly desirable in order to cover reactions of both hard and soft nucleophiles. Mechanistic studies with appropriate analytical tools should be conducted in order to obtain more insight on the transfer of the electrophilic CF_3_ group. A bimolecular nucleophilic substitution, S_N_2 type mechanism, is often suggested, although a single electron transfer mechanism cannot be ruled out depending on the reagent and reaction conditions. Of special interest is the question of asymmetric trifluoromethylation: Is it possible to induce stereoselectivity by electrophilic trifluoromethylation with the aid of an optically active trifluoromethylating reagent? Both our research groups are currently attempting to provide an answer to this question. It is the authors’ hope that this review will stimulate chemists to conduct further research that lead to the design of new reagents and to optimize the present ones for selective electrophilic trifluoromethylation of a wider range of substrates.
